# Variability of Lipids in Human Milk

**DOI:** 10.3390/metabo11020104

**Published:** 2021-02-11

**Authors:** Jayashree Selvalatchmanan, A.V. Rukmini, Shanshan Ji, Alexander Triebl, Liang Gao, Anne K. Bendt, Markus R. Wenk, Joshua J. Gooley, Federico Torta

**Affiliations:** 1 Singapore Lipidomics Incubator, Life Sciences Institute, National University of Singapore, Singapore 119077, Singapore; E0285343@u.nus.edu (J.S.); lsijish@nus.edu.sg (S.J.); triebl.alexander@gmail.com (A.T.); anne.bendt@nus.edu.sg (A.K.B.); 2 Department of Biochemistry, Yong Loo Lin School of Medicine, National University of Singapore, Singapore 119077, Singapore; gaoliang@nus.edu.sg (L.G.); bchmrw@nus.edu.sg (M.R.W.); 3 Neuroscience and Behavioral Disorders Program, Duke-NUS Medical School, Singapore 169857, Singapore; rukmini.dhara@duke-nus.edu.sg

**Keywords:** lipids, lipidomics, mass spectrometry, human milk, biological variation

## Abstract

Lipids in breastmilk play a critical role in infant growth and development. However, few studies have investigated sources of variability of both high- and low-abundant milk lipids. The objective of our study was to investigate individual and morning–evening differences in the human milk lipidome. In this study, a modified two-phase method (MTBE: Methanol 7:2) was validated for the extraction of lipids from human breastmilk. This method was then applied to samples from a group of 20 healthy women to measure inter- and intra-individual (morning versus evening) variability of the breastmilk lipidome. We report here the levels of 237 lipid species from 13 sub-classes using reversed-phase liquid chromatography mass spectrometry (RP-LCMS) and direct-infusion mass spectrometry (DI-MS). About 85% of lipid species showed stable inter-individual differences across time points. Half of lipid species showed higher concentrations in the evening compared with the morning, with phosphatidylethanolamines (PEs) and triacylglycerols (TAGs) exhibiting the largest changes. In morning and evening samples, the biological variation was greater for diacylglycerols (DAGs) and TAGs compared with phospholipids and sphingolipids, and the variation in DAGs and TAGs was greater in evening samples compared with morning samples. These results demonstrate that variation in the milk lipidome is strongly influenced by individual differences and time of day.

## 1. Introduction

Milk is the primary source of nutrition for infants during early development. Human milk (HM) contains an array of molecules responsible in aiding digestion and absorption of nutrients, assisting development, and maturation of vital organs/systems, and conferring immunological protection [1,2,3]. Given the importance of HM for child growth and brain development, there have been numerous efforts to identify the constituents of breastmilk. The primary contents of HM are carbohydrates (7%), lipids (5%), and proteins (0.9%), followed by lesser amounts of minerals, hormones, vitamins, and immune factors [4]. There are multiple sources of variation in HM, including dietary intake, the mother’s metabolic status (e.g., healthy versus diabetic), time within feeding, time of day and lactation stage [5,6,7]. Lipids supply about 50% of an infant’s energy requirements and serve as structural building blocks for brain and tissue development, and support gastrointestinal functions [8]. Lipids in HM are packaged in globules; these globules contain a triacylglycerol (TAG)-rich core, encapsulated by a triple-layered membrane, also known as the milk fat globular membrane (MFGM). The lipid portion of the MFGM is rich in phosphatidylethanolamines (PE), phosphatidylcholines (PC), sphingomyelins (SM), phosphatidylserines (PS), phosphatidylinositols (PIs), and cholesterol, but also contains small amounts of other bioactive lipids, such as gangliosides, lysophospholipids, monohexosyl- (Hex1Cer), and dihexosylceramides (Hex2Cer) [9]. For example, the relative abundance of SM in HM is high and may be linked to the amount of myelination required in the central nervous system (CNS) of the growing infant. Sphingomyelin, together with PC, is also a source of choline, which is important for development of the CNS [10].

Although HM provides all nutritional requirements for infant development, many lactating mothers do not breastfeed due to health-related issues, difficulties in breastfeeding or lifestyle choices [11]. In such cases, HM is often supplemented or replaced by donor milk or formula milk. In principle, supplementary sources of milk should reflect as closely as possible HM and its composition during breastfeeding. It is, therefore, crucial to measure the components of milk in-depth [12] (in this case, the lipidome) and understand, in more detail, sources of variability. This information would not only enable development of more biologically realistic formula milk, but may also provide key health-related information on factors that modulate the composition of donor milk.

### 1.1. Analytical Challenge of Human Milk Lipidomic Analysis

The lipid constituents of HM are very diverse in terms of polarity and relative concentration. The most abundant components (98% of the total) are the highly hydrophobic triacylglycerols (TAGs), while only 1–2% is represented by polar lipids, such as PE, PC, PI, SM, PS, lysophosphatidylcholine (LPC) and lysophosphatidylethanolamine (LPE) [13,14]. The obvious contrast in the physicochemical properties of these metabolites calls for different extraction approaches, which are essential to increase the lipidome coverage, but may require a longer sample preparation time and can sometimes become tedious.

A second challenge is that the high abundance of neutral lipids requires an extensive dilution of the extract before injection into the mass spectrometer; this prevents saturation of the detector and suppression of ionization. On the other hand, since phospholipids are of such low abundance, they require efficient enrichment before analysis. Concentrating the sample without removal of TAGs would cause signal suppression of the less abundant species, resulting in unreliable measurements. In this case, solid-phase extraction (SPE) methods can be used to separate the neutral lipids from the polar ones. However, such methods are time-consuming and more expensive when compared to liquid-liquid extraction (LLE) techniques. It is challenging to find a single extraction (or analytical) method which can comprehensively be applied to a broad spectrum of lipids in HM. As a result, several methodological papers have recently been published to circumvent some of the challenges described [15,16,17,18].

### 1.2. Diurnal Variation of Breast Milk Components

Lipid synthesis, storage, and transport are regulated by the circadian (“around a day”) system [19]. Given that rest-activity, feeding, and the plasma lipidome are diurnally regulated by the circadian clock and environmental factors [20], the composition of HM may likewise vary by time of day. Several studies have investigated diurnal variation of total fat content in HM using different methods of collection including pre-/post-feed expressed samples [21,22,23], total milk from a fully expressed breast [24,25,26,27,28], and demand feeding versus scheduled samples. Despite different sampling approaches, it has been consistently observed that total milk fat exhibits a 24-h rhythm, with most studies finding higher concentrations in the evening compared with other times of day [29]. Diurnal variation has also been observed for other milk components, including amino acids, minerals, and melatonin [30,31,32,33]. These studies suggest that the circadian system regulates the composition of milk and may serve as an important source of intra-individual variance. While prior studies have reported diurnal variation of total TAG levels, cholesterol, and cortisol in milk [29], there is almost no information on diurnal regulation of low abundant lipids, e.g., sphingolipids or phospholipids.

### 1.3. Aims and Objectives

From a compositional point of view, human milk is a complex and highly dynamic biofluid. While there is evidence of diurnal variation in HM, there are contradictory findings regarding the time course of lipid levels. Additionally, prior studies have focused predominantly on either total fat content [25] or TAG levels [6]. Recent work suggests that the milk fat globular membrane (MFGM) content of low-abundant bioactive lipids may confer benefits on infant development [10,34]. It is therefore important to investigate sub-classes of phospholipids (PLs) and sphingolipids (SPs) in breastmilk. Measuring the lipid composition of breastmilk is important for relating individual differences in HM with health outcomes, including infant growth, metabolism, and neurodevelopment. With this long-term goal in mind, our first objective was to validate a fast 2-phase MTBE/MeOH LLE method to analyze hundreds of lipid species from 10 μL of milk. Our second objective was to apply this method to study between-individual differences and within-individual differences in HM lipids from milk samples collected in the morning (06:00 to 09:00) versus the evening (18:00 to 21:00).

## 2. Results

### 2.1. Optimization of the Analytical Workflow

**Figure 1:**
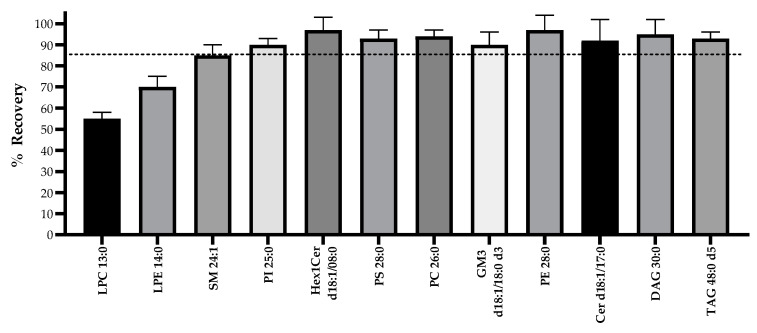
Recovery of 12 lipid standards from different classes when using a modified MTBE/methanol 2-phase extraction method. Error bars represent standard deviation (*n* = 6). The dotted line indicates the average recovery (88%).

Lipid extraction was performed according to a modified version of the two-phase extraction reported previously by Matyash and colleagues [35]; this method is commonly used for plasma and tissues but, to the best of our knowledge, has not been previously applied to human milk samples. An advantage of using this extraction over the conventional two-phase Bligh and Dyer [36] or Folch [37] methods is that the organic layer (and the lipids dissolved in it) containing methyl-tert-butyl ether/methanol is less dense than water and sits at the top, making this protocol a good candidate for automation when considering large-cohort analyses. This also prevents any contamination with aqueous components that may be present in milk when the extract is retrieved. The extraction protocol is detailed under Section 4.3—Lipid Extraction. The recovery of 12 lipid standards spiked in HM using this method was >85% for all of the classes, except for the lysophospholipids (Figure 1). Although the recoveries of the lysolipids were not optimal, they were still reproducible, as shown by a percentage coefficient of variation (%CV; *n* = 6) of 5% and 7% for LPC, and LPE, respectively.

The linearity of measurements was also tested to ensure reliable relative quantification of the different lipid species. This is especially relevant for the analysis of HM samples, as big differences exist between the concentrations of the neutral lipids, and phospholipids and sphingolipids. An R^2^ value of 0.85 was set as the cut-off for the linear regression model and all of the endogenous lipids that showed an R^2^ > 0.85 within the quality control (QC) were included in the final dataset. Figures S1a–f and S2a–f show the linearity curves of representative lipids from each class.

**Figure 2:**
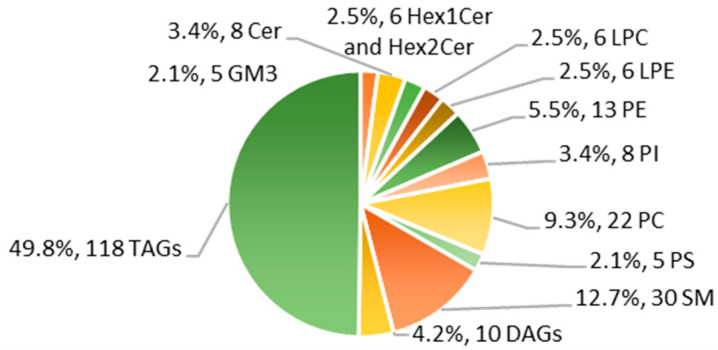
Number of lipids quantified in each class and relative percentage of total number of lipids reported via targeted and untargeted approaches in this study of human milk (HM).

We measured more than 380 lipids in HM, using both targeted and untargeted approaches. After multiple levels of QC filtering (See Section 4.5 Validation/Quality Control), we report a final number of 237 lipids from 13 sub-classes (Figure 2). A total of 109 species of PLs and SPs were quantified using reversed-phase liquid chromatography tandem mass spectrometry (RP-LCMSMS). An aliquot of the extract that was used for RP-LCMSMS was diluted ten times further before measuring 128 species of TAGs and DAGs with direct-infusion mass spectrometry (DI-MS). Figures S3 and S4 show a representative spectrum of the TAGs and DAGs analyzed via DI-MS and a representative chromatogram separating the various PL and SP classes using RP-LCMSMS. The total numbers of lipid species reported in each class are displayed in Figure 2.

### 2.2. Variation in the Milk Lipidome between Participants and within Participants

**Figure 3:**
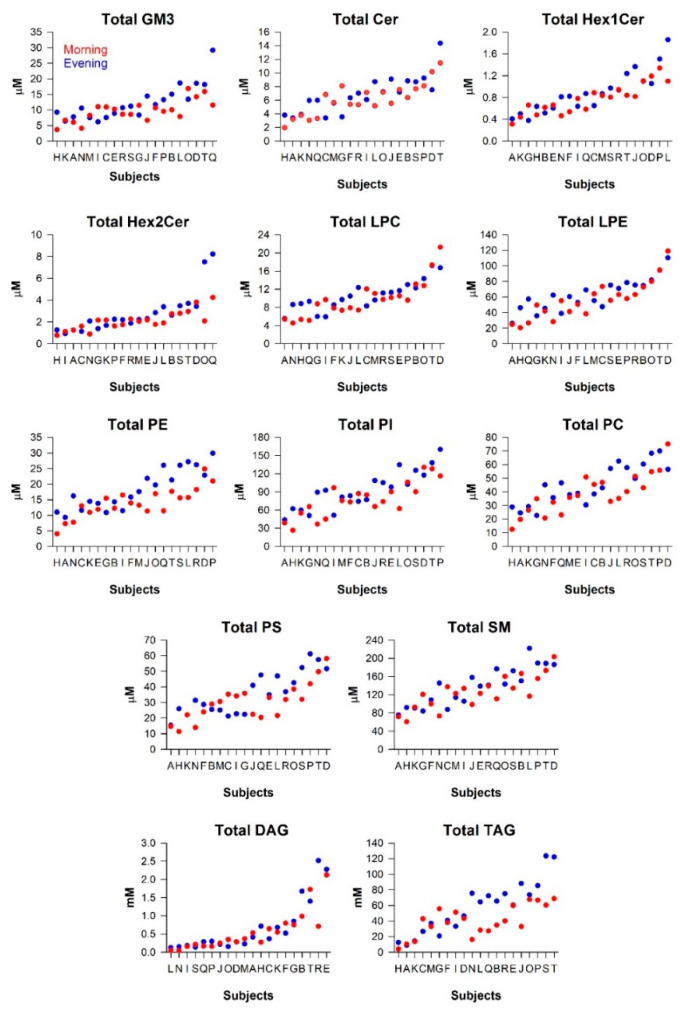
Individual differences in concentrations of human milk lipids. Lipid concentrations in morning (red) and evening (blue) milk samples are shown for 20 women. The data in each plot are ordered from left to right by the average concentration values of morning and evening samples in each participant.

Milk samples were collected from 20 participants in the morning (06:00 a.m.–09:00 a.m.) and evening (6:00 p.m.–9:00 p.m.), with the order of sample time randomized and counterbalanced (details under Section 4: Materials and Methods). Linear mixed models were used to test the stability of between-participant differences in milk lipids, and to test within-participant differences in morning versus evening milk samples (Table 1; Figure 3). The strength of individual differences was measured using the intraclass correlation coefficient (ICC), which represents the proportion of total variance attributed to between-participant variance (see Materials and Methods, Section 4.7 Data Analysis). We observed substantial individual differences in total Cers (ICC = 0.61, *p* = 0.012), Hex1Cers (ICC = 0.68, *p* = 0.007), LPCs (ICC = 0.70, *p* = 0.006), LPEs (ICC = 0.72, *p* = 0.006), and DAGs (ICC = 0.74, *p* = 0.005), and moderate individual differences in total Hex2Cers (ICC = 0.46, *p* = 0.034), PEs (ICC = 0.55, *p* = 0.018), PIs (ICC = 0.56, *p* = 0.017), PCs (ICC = 0.53, *p* = 0.020), PSs (ICC = 0.49, *p* = 0.027), SMs (ICC = 0.49, *p* = 0.029), and TAGs (ICC = 0.48, *p* = 0.029). Several lipid classes showed significant time-of-day variation with higher concentrations in the evening for total GM3s (*p* = 0.035), PEs (*p* = 0.002), PIs (*p* = 0.027), and TAGs (*p* = 0.011). There was no effect of order of HM collection (morning first versus evening first) on lipid concentrations.

**Figure 4:**
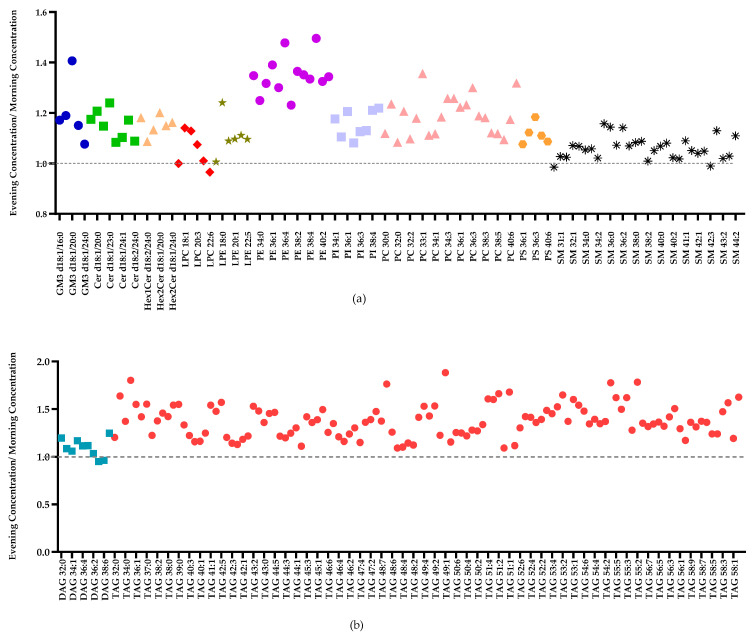
(**a**) The observed median fold-change for PLs and SPs; (**b**) The observed median fold-change for DAGs and TAGs. (Note: For better visual clarity, every lipid class is represented by a different colour, and only one of every two lipid species (symbols) is labelled on the x-axes. See Tables S1 and S2 for full list of values.)

Next, we investigated between- and within-participant differences using variance components analyses for individual lipid species. There were 13 lipid species that showed “almost perfect” reproducibility of individual differences (ICC > 0.80, *p* < 0.003), including LPE 22:1, SM 44:2, PE 40:2, SM 43:2, LPE 16:0, PE 38:1, PE 32:0, LPC 20:1, PE 38:2, DAG 36:3, LPE 20:1, DAG 36:2, and PE 34:1 (Table S4). An additional 90 lipid species exhibited substantial individual differences (ICC 0.60–0.80, *p* < 0.013), and 103 lipid species exhibited moderate individual differences (ICC 0.40–0.60, *p* < 0.05). Half of the lipid species examined (119 out of 237) showed evidence of time-of-day differences with higher concentrations in the evening relative to the morning (four lipids, *p* < 0.001; 11 lipids, 0.001 > *p* < 0.01; 104 lipids, 0.01 > *p* < 0.05) (Table S4). PEs and TAGs showed the strongest morning–evening differences in concentration levels, with the top 11 most affected lipids belonging to the PE class (PE 34:1, PE 32:2, PE 38:3, PE 38:2, PE 36:1, LPE 18:0, PE 40:7, PE 36:2, PE 38:6, PE 38:4, PE 36:4) (Figure 3 and Table S4). Several lipid species belonging to PIs, PSs, GM3s, and PCs also showed evidence of higher concentrations in the evening, whereas the majority of LPCs, DAGs, Cers, SMs, and LPEs did not show morning–evening differences. The data were further visualized by plotting the median fold-change (evening/morning) of each of these lipids (Figure 4a,b), demonstrating a trend for higher concentrations in milk lipids in the evening across all sub-classes.

### 2.3. Inter-Individual Variation in the Human Milk Lipidome in the Morning and Evening

**Figure 5:**
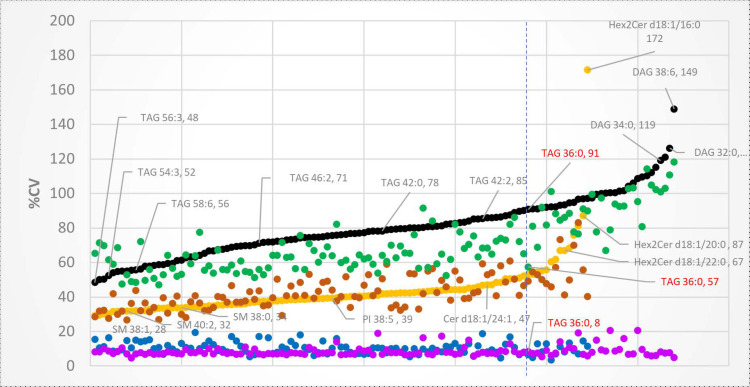
Biological variation, represented as percentage coefficient of variation (%CV), of lipids in human milk. Lipid classes are represented by different colours. Triacylglycerols and Diacylglycerols - Evening (Black), Morning (Green), Phospholipids and Sphingolipids – Evening (Yellow), Morning (Orange). The %CV of technical quality controls (QC) of Triacyclglycerols and Diacylglycerols (Purple) and Phospholipids and Sphingolipids (Blue) are shown for comparison. The vertical dotted line shows, as an example, the position and the %CV values of TAG 36:0 in the evening, in the morning and in the QC samples. All the lipids are plotted from the lowest to the highest %CV (sorted according to evening samples).

Inter-individual variation for each lipid species was examined separately for morning and evening samples by calculating the biological coefficient of variation (%CV). At both time points, neutral lipids exhibited a much higher biological variation than PLs and SPs (except for Hex2Cer d18:1/16:0) (Figure 5). In addition, the inter-individual variation for the NLs was generally lower in the morning, whereas that of the PLs and SPs was similar between morning and evening.

Table 2 and Table 3 show the biological and technical variations of the 20 most and least variable lipids in HM found in this study. TAGs and DAGs were the most variable species; we observed that 90% of the measured DAGs were among the 20 most variable lipids in the morning (Table 2), while 60% of them were among the 20 most variable lipids in the evening. The SMs, on the other hand, displayed low variability across the 20 women sampled. Of the 22 SMs measured, 68% of them were shown to be among the least variable in the morning, while 45% of them were shown to be among the 20 least variable lipids in the evening together with few PC species (Table 3).

## 3. Discussion

### 3.1. Summary of Findings

In the present study, we validated a novel analytical workflow for extracting and quantifying lipids in HM. Using a MTBE/MeOH extraction method followed by RP-LCMSMS and DI-MS, we were able to measure the concentrations of 237 lipid species from 10 μL of breastmilk. This approach was used to analyze between- and within-participant variation in milk lipids. We found the between-participant differences were moderate-to-substantial for most lipid sub-classes and species, demonstrating stability of individual differences for milk samples collected at different time points. Additionally, morning–evening differences in milk lipids were observed for nearly half of all lipid species, with higher concentrations observed in the evening compared with the morning. In particular, the concentrations of PEs and TAGs were nearly 1.5-fold higher in the evening. Lastly, we showed that biological variation (%CV) was higher for DAGs and TAGs compared with phospholipids and sphingolipids, and variation of DAGs and TAGs was generally higher in evening samples relative to morning samples. Together, these findings demonstrate that variation in milk lipids is influenced strongly by individual differences and time of day.

### 3.2. Optimization of Analytical Workflow

Several lipid extraction methods, previously validated in other publications, were tested on HM at the beginning of our study (data not shown). We tested single-phase extraction methods, such as butanol/methanol (BuMe 1:1) [38], butanol/methanol (BuMe 3:1) [18], and butanol/methanol/chloroform (BuMeCh3Cl 3:5:4) [18], together with the two-phase MTBE/MeOH [35] method. In our hands, the MTBE/MeOH extraction, which had not previously been validated for use with HM, had benefits that outweighed those offered by other extractions and was the one we selected for all our lipidomic analyses of HM.

One crucial point in favor of the above-mentioned method was the lack of precipitation observed in the extracts upon storage; the presence of precipitation is often an issue when single-phase lipid extractions are used with HM, due to the high concentration of neutral lipids. Indeed, precipitation after extraction has previously been reported for BuMe (1:1) and BuMe (3:1) solvent systems [18]. The butanol/methanol/chloroform (3:5:4) extraction introduced by Liu et al. [18] is more compatible with the neutral lipid levels in HM. However, when drying the supernatant to concentrate the sample, we observed the formation of a white residue that could not be resolubilized in chloroform/methanol (1:1) or BuMeCh3Cl (3:5:4). As precipitation issues can cause unreliable analytical measurements and clogging of LC systems, we decided to try alternative methods for lipid extraction. Single-phase extraction methods, although efficient for human plasma [38], often cause precipitation issues when used on human milk samples and must be chosen carefully according to the user’s intended workflow (drying, extended storage etc.).

Another factor in favor of the MTBE/MeOH extraction is that this workflow is compatible with automated sample preparation, required for large cohort studies. The organic supernatant containing lipids lies in the upper phase of the solvent system (unlike in the Bligh and Dyer or Folch methods) and is more easily retrieved by robotic systems without disturbing the phase separation. Another benefit of this method is the preparation time, which is shorter than conventional solid-phase extraction-based techniques, used to separate the highly abundant TAGs from the PLs. Moreover, many of the previously published methods require a large starting volume of milk [15], whereas we used only 10 μL to quantify over 200 lipids.

Direct infusion (DI) of lipids into the mass spectrometer was initially explored as a fast and efficient method for analyzing the milk lipidome. This shotgun lipidomic analysis is based on infusion with a nano-source and the acquisition of high-resolution MS and MS/MS spectra for all potential lipid species using a Q-Exactive Orbitrap [12]. However, the effect of ion suppression, probably due to the presence of TAGs, was extremely high. The resulting phospholipid/sphingolipid signals were often too low and inconsistent (data not shown). DI of diluted samples into a high-resolution mass spectrometer was therefore the method of choice for TAG and DAG analysis, while RP-LCMSMS (utilizing dynamic multiple reaction monitoring; dMRM) was the optimal solution to measure phospholipids and sphingolipids.

### 3.3. Variability of Lipids in Human Milk

#### 3.3.1. Inter-Individual Variation

Our study is the first to examine systematically the strength of individual differences for different lipid sub-classes and species. We found that between-participant differences were substantial for total Cers, Hex1Cers, DAGs, LPEs, and LPCs (ICC, range = 0.61–0.74), and moderate for total Hex2Cers, PEs, PIs, PCs, PSs, SMs, and TAGs (ICC, range = 0.46–0.56). At the level of individual milk lipids, the reproducibility of between-participant differences was almost perfect for 5.5% of lipid species (ICC, range = 0.80–0.94), substantial for 38.0% of lipid species (ICC, range 0.60–0.79), and moderate for 44.3% of lipid species (ICC, range = 0.40–0.60). Hence, the majority of lipid species analyzed in our study showed evidence of stable individual differences for milk samples collected at different times of day (86.9% of lipid species, ICC > 0.4 and *p* < 0.05). Our findings are consistent with prior studies in which inter-individual variation was greater than intra-individual variation for lipids in milk [23,39,40] and blood plasma [41]. For example, one study of 52 women found that the variability of the lipid content in milk was four times higher between individuals than within individuals [39]. Another study of six women reported that the cholesterol variation in milk was five times higher between individuals compared to variation within individuals [40].

Previous studies have also reported wide variation in the fat content of milk from different women, including the 24-h amounts of total fat [42]. In a study of 52 women who provided ~10 milk samples over a 24-h period, the %CV for TAGs in pooled samples was 22%, and the %CV ranged from 28% to 39% for samples collected at different time points [39]. In the present study, the %CV for the total concentration of all measured lipids was 49% in the morning and 57% in the evening. For 128 species of TAGs and DAGs, the biological variation ranged from 47% to 118% in the morning, and from 48% to 149% in the evening. Most TAGs and DAGs showed greater variation in the evening compared with the morning. By comparison, biological variation of the 109 PLs and SPs was similar at both time points and much lower compared with TAGs and DAGs, ranging from 27% to 83% in the morning, and 29% to 87% in the evening.

We found that SM was the least variable lipid class across our sample of 20 women. SM is present in the MFGM and is known to play an essential role in the development of the infant’s nervous system [43,44]. SM is also an important source of choline for the infant. In a recent nutritional intervention study, SM-fortified milk was found to improve the neurobehavioral development of low-weight infants [45]. These essential functions might require a tighter physiological control of SM levels, hence contributing to lower between-participant variation compared with other lipid classes.

The only outlier in our dataset was Hex2Cer d18:1/16:0, which had a %CV of 172% in the evening. This result was driven by one participant whose HM concentration of Hex2Cer d18:1/16:0 was much higher than other participants. The rest of the lipids from the same donor were within the normal range. Since dihexosylceramides are not commonly measured in HM, no previous data are available to suggest an explanation for this finding.

There are several possible sources of biological variation of lipids in our study. In addition to differences that may arise due to individual variation in genetics and physiology, lipids in HM are affected by lactation stage, time elapsed since last feed, the extent of breast emptying, hormonal changes [46,47], and maternal diet. Milk composition also varies during a feed, with foremilk having more water and less fat compared with hindmilk. Some studies have shown that the BMI of the mother is associated with the fat concentration of milk in both well-nourished and under-nourished populations [39,48]. In the present study, we attempted to minimize these sources of variance by recruiting women who were healthy and at a similar stage of lactation, and by asking participants to fully express one breast (i.e., pooling foremilk and hindmilk). The biological variation in our study is likely representative of healthy Singaporean women, but additional studies are needed to understand better the sources of biological variation of lipid species in breastmilk.

#### 3.3.2. Intra-Individual Variation

About half of lipid species measured in our study showed evidence of time-of-day variation. Consistent with prior studies of total fat [29], TAGs [39,49], and cholesterol [14,49,50] in human milk, we found that the concentrations of many species of glycerolipids, phospholipids, and sphingolipids were higher in the evening compared with the morning. By comparison, earlier studies have found that sterol lipids including cortisol and cortisone reach their peak in breastmilk in the morning [29], similar to the rhythm observed in blood plasma and saliva [19,51]. There is little evidence, however, that concentrations of free fatty acids exhibit diurnal variation in HM [29], which contrasts with results in blood plasma [52].

Nearly all studies that have investigated diurnal variation of HM lipids have focused on total fat, which predominantly comprises TAGs [39,53]. We extended previous work by demonstrating that more than 80 individual TAG species exhibited higher concentrations in the evening. In addition, our study is the first to investigate systematically morning-evening differences in low abundant lipids, such as phospholipids and sphingolipids, which play key roles in infant growth and development [9,10,45,54]. Among all of the lipids analyzed, PE species exhibited the largest change in concentrations between morning and evening samples. PE is an important component of cell membranes and they are mainly concentrated on the inner surface of the MFGM, together with PS and PI [55]. However, their specific function in human milk has not been elucidated.

Differences in the concentration of lipids in HM across time of day could be driven by circadian regulation of the mother’s mammary gland tissues, and/or by diurnal variation in behavioral factors, such as the timing of sleep and feedings in mother and child. In principle, an increase in milk water volume could lead to a lower percentage of fat in milk [14]. In a study of women who expressed milk from the same breast every 4 h over a 24-h period, milk volume was shown to exhibit diurnal variation with peak levels in the morning (08:00 –12:00), coinciding with lower total fat in HM [56]. Another important determinant of milk volume and lipid concentration is the time elapsed since the last feed (from that particular breast), with a longer interval leading to lower percentage of fat in milk [22]. In our study, the milk volume and time since last feeding did not differ between morning and evening samples. The higher concentration of lipids in evening milk could be attributed to postprandial effects, e.g., if the mothers consumed more food before the evening sample compared with the morning sample. However, unlike the postprandial increase of TAGs in blood, an increase in fat levels in milk immediately upon food consumption has not been demonstrated reliably [25,56]. Additionally, 11 out of 20 participants in our study had dinner before providing their evening milk sample (while only 2 out of 18 participants had breakfast before breastfeeding). The consumption of dinner before breastfeeding did not significantly affect the lipid composition in the samples as shown by the PCA plot in Supplementary Figure S5. Additional studies are needed to determine whether the source of diurnal variation in milk lipids is driven predominantly by the circadian clock or by other bio-behavioral factors.

Our findings have potential implications for milk research and infant development. We showed that each mothers’ milk had a unique lipid profile (i.e., there were stable individual differences), and the concentrations of lipids showed time-of-day variation. Given that breastmilk is the primary source of nutrition in infants, diurnal variation of milk may regulate diurnal physiology of the child including fat metabolism. Nutritional and metabolic signals are known to reset and synchronize circadian clocks. Hence, a mismatch in the timing of milk expression (e.g., stored milk or donor milk) and feeding could have implications for a child’s circadian patterns of metabolism and behavior. Similarly, formula milk is not designed to reflect natural diurnal variation in HM. Future studies should investigate individual differences in breastmilk lipids in different geographic and sociocultural settings, in populations comprising women of different nationalities and ethnic groups. Moreover, studies should investigate the implications of such individual differences and diurnal variation in breastmilk on infant health.

## 4. Materials and Methods

### 4.1. Participants and Recruitment

In a within-subjects study design, twenty healthy, non-obese women (25–35 years old) were recruited, between July and September, to provide two breast milk samples collected approximately 12 h apart. Due to its geographical location, Singapore experiences no distinct seasons, and for this reason, no seasonal effect on milk composition was considered. Participants were mothers of full-term babies from singleton pregnancies, no older than three months of age at the time of recruitment. The babies’ sole source of nutrition was breast milk, by either latching or pumping. All babies were born healthy, except one who was discovered to have a heart defect. Women were ineligible if they were smokers or were diagnosed with gestational diabetes or other chronic medical conditions. Individuals who were taking drugs or supplements intended to boost production of breast milk were also excluded. All study procedures were approved by the National University of Singapore Institutional Review Board. Participants provided written informed consent. Demographic characteristics of participants are presented in Table 4.

Eligible participants were asked to maintain a food diary and a breastfeeding diary for one week. Participants completed a meal diary over the 3-day period preceding milk collection, which was used to document the timing and types of meals that were consumed. However, meal portions were not recorded. Participants in our study consumed a predominantly Chinese–Singaporean diet (19 out of 20 women were Chinese–Singaporean). During sample collection, researchers went to the participants’ home to collect two breast milk samples, one in the morning (between 06:00 and 09:00) and the other in the evening (between 18:00 and 21:00). Participants started expressing the milk only after the researcher reached their home. The order of sample collection (morning first or evening first) was randomized and counterbalanced. Participants were asked to fully express milk from one breast using their own breast pump, at the time when they would normally breastfeed. The milk sample was mixed by gentle swirling and the researcher then collected 10 mL of the milk using a pipettor. The remainder of the milk was returned to the participant. Participants were asked to express milk from the same breast at both time points. Milk sample characteristics are presented in Table 5.

The 10-mL milk sample was immediately pipetted into 450 µL aliquots and stored on dry ice during transport. Samples were then stored at −80 °C (< 1 hour after collection) until they were analyzed. Prior to lipid analysis, each sample was subsequently subjected to an additional freeze-thaw in order to generate 10–20 µL aliquots. Pairwise randomization of samples was performed, and the order followed through from the extraction of samples to instrument run.

### 4.2. Chemicals for Sample Analysis

Acetonitrile, methanol, and 2-propanol (Optima LC-MS grade) were purchased from Fisher Chemical(Waltham, USA); methyl-tert-butyl ether (MTBE), ammonium formate, ammonium hydrogen carbonate (BioUltra grade) and chloroform (analytical reagent grade) were purchased from Sigma-Aldrich (St. Louis, USA) Deionized water was obtained from an in-house water purification system. The following standards, lysophosphatidylethanolamine (LPE) 14:0, Lysophosphatidylcholine (LPC) 13:0, phosphatidylcholine (PC) 13:0_13:0, phosphatidylethanolamine (PE) 14:0_14:0, phosphatidylserine (PS) 14:0_14:0, phosphatidylinositol (PI) 12:0–13:0, ceramide d18:1/17:0, glucosyl(ß) C8 ceramide, hexosyl1ceramide d18:1/8:0 and 06:0 sphingomyelin (SM) were purchased from Avanti Polar Lipids (Alabaster, USA). GM3 d18:1/18:0 d3 was purchased from Matreya LLC (State College, USA). Diacylglycerol (DG) 15:0_15:0 was purchased from Santa Cruz Biotechnology, Inc. (Dallas, USA). Glyceryl triheptadecanoate (TAG) 17:0_17:0_17:0 was purchased from Sigma Aldrich (St. Louis, USA) and glyceryl trihexadecanoate (TAG) 48:0 d5 was purchased from CDN Isotopes (Pointe Claire, Canada)

### 4.3. Lipid Extraction

A total of 180 μL of chilled ammonium bicarbonate (150 mM) and 810 μL of methyl-tert-butyl-ether/methanol (MTBE/MeOH 7:2) containing an internal standard mix (See Table S3) were added to 10 μL of human milk. Samples were vortexed for 30 s, sonicated for 30 min and centrifuged for 5 min at 3000 g. The upper layer (600 μL) was then transferred out to a clean tube, dried down and reconstituted in 200 μL Chloroform/methanol (1:1 *v/v*) (Portion A)

### 4.4. Mass Spectrometric Method

#### 4.4.1. Triacylglycerol and Diacylglycerol Measurements

A total of 50 μL of portion A were removed and diluted further with 450 μL of a mixture of isopropanol/methanol/chloroform (4:2:1 *v*/*v*) containing 7.5 mM ammonium formate. The samples were subsequently measured in positive mode via direct-infusion mass spectrometry (DI-MS) on a TriVersa NanoMate (Advion Biosciences, Ithaca, NY, USA) coupled to a Thermo QExactive plus quadrupole-orbitrap mass spectrometer. Samples were kept in a 96-well plate at 4 °C for the duration of the entire run. Samples in the well were taken up by nanotips and sprayed through nano-electrospray nozzles present on an electrospray ionization (ESI) chip into the MS. Each individual tip and nozzle were used only once, eliminating concern of contamination/carryover. Once the spray was initiated from the NanoMate, a period of 30 s was allowed for spray stabilization before the acquisition of data, which was set to be within the mass range of 400 to 1050 *m/z*. The precursor scans were acquired for one minute at a resolution setting of 140,000 and the subsequent product ion scans were acquired for 1.1 min at a resolution setting of 17,500 with an inclusion list containing all of the masses from 400 to 1050 with 1Da intervals. Results were processed using LipidXplorer 1.2.7, a common open-source software for shotgun data. Lipids were identified by their accurate mass (tolerance of up to 5ppm for MS1) and tandem MS spectra.

#### 4.4.2. Phospholipids and Sphingolipids Measurements

Portion A of the extract was measured in positive mode electrospray ionization (ESI) mass spectrometry (MS). The lipids were measured via reversed-phase chromatography with a 1290 infinity UHPLC coupled to an Agilent 6495A QQQ system. An Agilent Zorbax RRHD Eclipse C18 column (2.1 × 50 mm) was used for separation of the different phospholipid classes. A dynamic multiple reaction monitoring system (dMRM) was used to monitor both the precursor and product ions transitions for each lipid. Identities of peaks were dependent on retention time (RT) and the specific MRM transitions for each lipid. Raw peak areas were generated using Agilent MassHunter Quantitative Analysis software Version 8 (Agilent Technologies, Santa Clara, USA). The M+3 isotopic effect of SM X:Y on PC X-4:Y-1 and M+3 isotopic effect of GM3 d18:1/18:0 on GM3 d18:1/18:0 d3 (internal standard, ISTD) were both corrected. The peak areas of the endogenous lipids were then normalized to the areas of the internal standards (specific for each class).

### 4.5. Method Validation/Quality Control

To ensure that the method used in conjunction with both analytical platforms was reliable, a linearity determination was performed by extracting varying initial volumes of samples with a fixed amount of ISTD. These varying volumes corresponded to 40%, 60%, 80%, 100% 120%, and 160% of the standard volume of milk that was used for extraction in this study (10 μL corresponding to 100%). Moreover, 560 μL of ammonium bicarbonate were added to 140 μL of a pooled milk sample. This diluted sample was then split into different volumes—each sample volume being analyzed in duplicate—and used to investigate the response of all of the endogenous lipids measured on both analytical platforms.

A collection of pooled Quality Controls was added to the extraction and instrument worklists. Technical quality controls (TQCs), which are replicate injections of a pooled extract, were injected every 12 study samples. Batch quality controls (BQCs), extracted independently from a pooled sample and which measure both extraction and instrument variations, were injected every six samples over the duration of the worklist. Extraction blanks were also inserted at the start and at the end of the analysis.

Finally, only the lipids adhering to the following QC criteria were quantified: (1) coefficient of variation for both TQCs and BQCs < 20%; (2) S/N > 10; (3) calculated R^2^ for linearity >0.85.

### 4.6. Internal Standards

Eleven internal standards pertaining to the respective class of lipids measured were used for normalization and estimation of relative concentrations. DAGs were normalized to TAG 48:0 d5 and dihexosylceramides (Hex2Cer) were normalized to Glucosyl C8 Ceramide (Hex1Cer d18:1/8:0). All other lipids were normalized to standards from their respective classes. The standards were spiked into the MTBE/MeOH (7:2) solvent mixture, which was then used to extract the lipids from milk. Internal standard concentrations are detailed in Table S3.

### 4.7. Data Analysis

A missing values imputation using quantile regression imputation of left-censored (QRILC) data was performed on four data points that had missing values.

Linear mixed models were used to estimate between-participant and within-participant variances [57,58] for different lipid sub-classes and species. Models included a normally distributed random intercept to represent between-participant differences around a fixed intercept, and a normally distributed random error for within-participant differences. Fixed effect corrections were performed for time of day (morning, evening) and order (morning first, evening first) in which milk samples were collected. Linear mixed models were evaluated using the restricted maximum likelihood method. Reproducibility of between-participant differences were quantified using the intraclass correlation coefficient (ICC). For each lipid, the total variance was partitioned into between- and within-participant variance (σ2BS and σ2WS, respectively), correcting for fixed effects. The ICC was calculated as ICC = σ2BS/(σ2BS + σ2WS), representing the proportion of overall variance attributed to individual differences. The ICC values, which can range from 0 to 1, were interpreted according to the following benchmark ranges [59]: “moderate” (0.4–0.6), “substantial” (0.6–0.8), and “almost perfect” (0.8–1.0). Statistical significance of the ICC was assessed by a Wald Z-test of the between-participant variance, and fixed effects were tested using an F-test. The threshold of significance was set as *p* < 0.05.

Biological variation (% CV) of each lipid = (standard deviation of lipid concentration across 20 samples/Average concentration of lipid) *100%.

## Figures and Tables

**Table 1:** Variance components analysis for human milk lipids. Z: Wald Z-test value *p*: *p*-value. (Significance set to 0.05), ICC: intra-class correlation coefficient, F: F-test value.

	Variance Analysis			Time of Day		Order of Sample	
**Lipid class**	**Z**	***p***	**ICC**	**F**	***p***	**F**	***p***
GM3	1.43	0.076	0.35	5.21	0.035	1.90	0.19
Cer	2.27	0.012	0.61	1.66	0.214	1.46	0.24
Hex1Cer	2.45	0.007	0.68	3.85	0.065	0.78	0.39
Hex2Cer	1.83	0.034	0.46	3.78	0.068	0.00	0.99
LPC	2.49	0.006	0.70	1.17	0.293	0.33	0.57
LPE	2.54	0.006	0.72	3.26	0.088	0.66	0.43
PE	2.10	0.018	0.55	13.72	0.002	1.48	0.24
PI	2.13	0.017	0.56	5.81	0.027	1.25	0.28
PC	2.04	0.020	0.53	4.15	0.057	1.62	0.22
PS	1.92	0.027	0.49	3.84	0.066	0.85	0.37
SM	1.90	0.029	0.49	2.35	0.143	0.56	0.47
DAG	2.59	0.005	0.74	1.30	0.270	1.45	0.24
TAG	1.89	0.029	0.48	7.95	0.011	1.18	0.29

**Table 2:** The twenty most variable lipids across all classes measured.

20 Most variable lipids					
Lipid	Biological Variation (%) Morning	Technical Variation % (QC)	Lipid	Biological Variation (%) Evening	Technical Variation % (QC)
DAG 38:6	118	5	Hex2Cer d18:1/16:0	172	11
TAG 47:0	114	7	DAG 38:6	149	5
DAG 32:0	111	8	DAG 32:0	126	8
DAG 34:2	105	6	DAG 36:1	121	8
TAG 43:0	103	7	DAG 34:0	119	8
DAG 36:1	103	8	DAG 34:1	115	6
TAG 45:0	103	7	DAG 34:2	112	6
DAG 34:1	102	6	TAG 47:0	110	7
DAG 36:4	101	6	TAG 32:0	110	9
DAG 34:0	101	8	TAG 42:5	109	21
TAG 36:2	99	8	TAG 43:0	106	7
TAG 38:3	97	9	TAG 45:0	104	7
TAG 42:5	95	21	TAG 50:8	101	16
TAG 41:0	94	9	TAG 34:1	101	10
TAG 50:8	93	16	TAG 41:0	100	9
TAG 34:1	92	10	TAG 37:0	100	21
DAG 36:3	92	5	TAG 34:0	100	9
TAG 60:3	92	16	TAG 38:3	100	9
TAG 49:1	91	7	TAG 44:6	100	15
DAG 36:2	90	6	TAG 36:2	97	8

**Table 3:** The twenty least variable lipids across all classes measured.

20 Least Variable Lipids					
Lipid	Biological Variation (%) Morning	Technical Variation % (QC)	Lipid	Biological Variation (%) Evening	Technical Variation % (QC)
SM 40:2	27	15	SM 39:1	29	16
SM 40:1	27	11	SM 44:1	29	10
SM 38:1	28	16	SM 36:0	30	11
SM 38:2	28	11	SM 38:1	30	16
SM 39:1	28	16	PI 36:1	32	9
SM 40:0	29	11	PE 38:4	32	8
SM 38:0	30	13	SM 41:1	32	9
PE 38:4	30	8	SM 40:2	32	15
PC 38:4	31	12	PC 34:2	32	11
GM3 d18:1/22:0	31	15	LPC 18:1	32	15
SM 41:1	32	9	SM 36:1	32	14
SM 44:1	32	10	LPC 22:5	32	15
SM 37:1	32	12	GM3 d18:1/22:0	33	15
SM 36:1	32	14	SM 34:1	33	10
SM 40:3	32	15	SM 40:1	33	11
SM 36:0	32	11	PC 32:0	34	7
Hex1Cer d18:2/22:0	32	8	PC 38:4	34	12
LPE 18:0	33	7	PC 34:0	34	10
SM 42:1	34	10	PC 34:1	34	8
SM 34:1	34	10	SM 38:0	34	13

**Table 4:** Participant characteristics (*n* = 20).

Characteristic	Mean (SD)	Range
Maternal age (y)	29.80 (2.63)	25–35
Maternal height (m)	1.60 (0.05)	1.53–1.73
Maternal weight (kg)	56.58 (6.43)	46–72
Maternal BMI	22.01 (2.00)	19.40–27.43
Gestational age of baby at delivery (weeks)	39.15 (0.75)	38–40
Age of child (days)	86.40 (8.23)	62–101
Birth weight of baby (kg)	3.19 (0.33)	2.44–3.86
Pre-pregnancy weight of mother (kg)	52.23 (6.00)	43–65
	**Number (%)**	
Ethnicity	19 Chinese (95), 1 Malay (5)	
Delivery mode (Vaginal)	15 (75)	
Sex of child (Female)	10 (50)	

**Table 5:** Milk sample characteristics.

	Evening		Morning		*p* Value
	Mean (SD)	Range	Mean (SD)	Range	
Total volume of breast milk expressed (mL)	70.58 (31.53)	25–130	84.45 (52.79)	20–225	0.10
Time taken for milk expression (h:mm)	0:17 (0:06)	0:07–0:33	0:18 (0:06)	0:08–0:34	0.31
Gap between previous feed and sample (h:mm)	2:07 (1:10)	0:13–4:15	2:17 (1:56)	0:18–9:07	0.75
Number of feeds between first and second sample	4.50 (1.50)	2–8	4.15 (1.31)	2–7	0.35
Number of participants who had dinner before evening sample, N (%)	11 (55)				

## Data Availability

The data presented in this study are openly available on Zenodo at http://doi.org/10.5281/zenodo.4471248.

## Supplementary Materials

The following are available online at https://www.mdpi.com/2218-1989/11/2/104/s1, Figures S1 (a,b): linearity curves for TAG 52:2 and DAG 34:1 extracted via 2-phase MTBE/MeOH method using 4–16 µL of human milk (analyzed using Thermo QExactive plus quadruple-orbitrap mass spectrometer). Figures S1 (c–f): linearity curves for PE 38:1, PI 36:2, SM 40:1 and PC 36:1 extracted via 2-phase MTBE/MeOH method using 4–16 µL of human milk (analyzed using Agilent 6495A QQQ). Figures S2 (a–f): linearity curve for GM3 d18:1/20:0, Hex1Cer d18:2/24:0, Hex2cer d18:1/16:0, LPC 18:1, LPE 18:1, Cer d18:1/24: extracted via 2-phase MTBE/MeOH method using 4–16 µL of human milk (analyzed using Agilent 6495A QQQ). Figure S3: representative spectrum of TAGs and DAGs in human milk via direct-infusion mass spectrometry. Full scan of precursors shown measuring from 400 to 1050 *m/z*. Figure S4: representative chromatogram separating the various PL and SP classes via RP-LCMSMS (using dMRM). Figure S5: PCA plot of participants who provided a milk sample before (0) and after (1) dinner. Table S1: evening/morning concentration (median) values for every PL and SL measured. Table S2: evening/morning concentration (median) values for every TAG and DAG measured. Table S3: list of class-specific internal standards (ISTDs) made up in extraction solvent (MTBE/MeOH 7:2) and their final concentrations in the measured extract. Table S4: variance components analysis of individual lipids. Table S5: full list of concentrations of lipids in every sample.
