# Supplementary material for: Variability of Lipids in Human Milk
Source: Metabolites. 2021 Feb 11;11(2):104. doi: 10.3390/metabo11020104 (PMC7916976; doi:10.3390/metabo11020104)
Supplement: Supplementary file 1 [file metabolites-11-00104-s001.zip › metabolites-1087212-supplementary_editingafterreview.docx]

Supplementary Materials: Variability of Lipids in Human Milk

Jayashree Selvalatchmanan^1,2^, A.V. Rukmini^3^, Shanshan Ji^1^, Alexander Triebl ^1,2^, Liang Gao^2^, Anne K Bendt^1^, Markus R Wenk^1,2^, Joshua J Gooley^3,^* and Federico Torta^1,2,^*

^1^Singapore Lipidomics Incubator, Life Sciences Institute, National University of Singapore, Singapore

^2^ Department of Biochemistry, Yong Loo Lin School of Medicine, National University of Singapore, Singapore

^3^ Neuroscience and Behavioral Disorders Program, Duke-NUS Medical School, Singapore

*****Correspondence: joshua.gooley@duke-nus.edu.sg; +65-6516-7430 (J.J.G.) [bchfdtt@nus.edu.sg](mailto:bchfdtt@nus.edu.sg) (F.T.)

**Figures S1 (a) and (b)** Linearity curves for TAG 52:2 and DAG 34:1 extracted via 2-phase MTBE/MeOH method using 4-16 µL of human milk (analysed using Thermo QExactive plus quadruple-orbitrap mass spectrometer)

**Figures S1 (c) –(f):** Linearity curves for PE 38:1, PI 36:2, SM 40:1 and PC 36:1 extracted via 2-phase MTBE/MeOH method using 4-16 µL of human milk (analysed using Agilent 6495A QQQ)

**Figures S2 (a)-(f):** Linearity curve for GM3 d18:1/20:0, Hex1Cer d18:2/24:0, Hex2cer d18:1/16:0, LPC 18:1, LPE 18:1, Cer d18:1/24: extracted via 2-phase MTBE/MeOH method using 4-16 µL of human milk (analysed using Agilent 6495A QQQ)


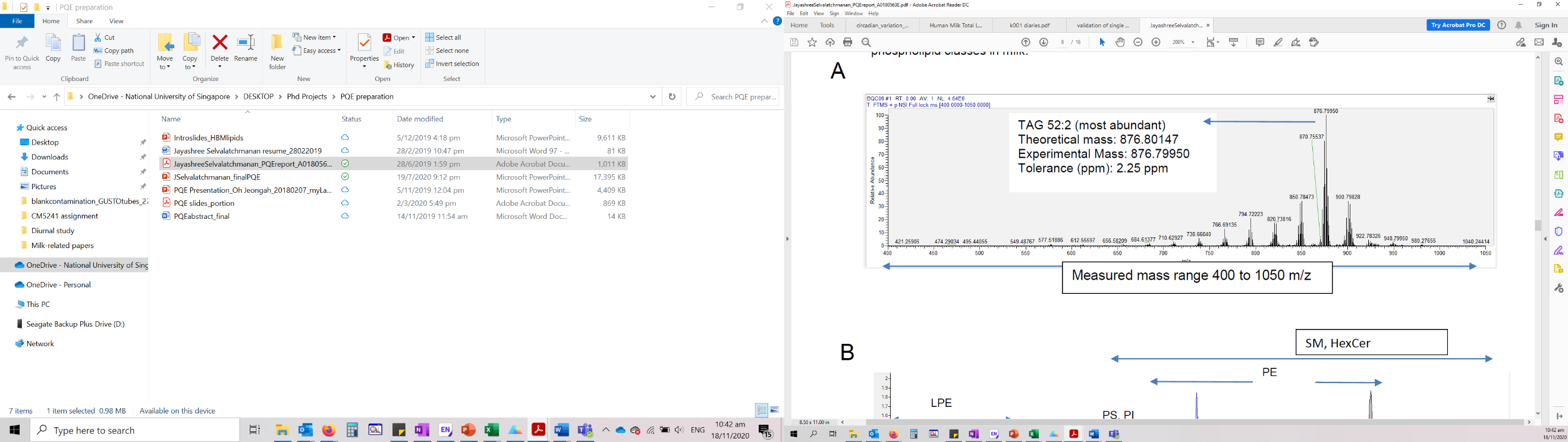


**Figure S3.** Representative spectrum of TAGs and DAGs in human milk via direct-infusion mass spectrometry. Full scan of precursors shown measuring from 400 to 1050 m/z.


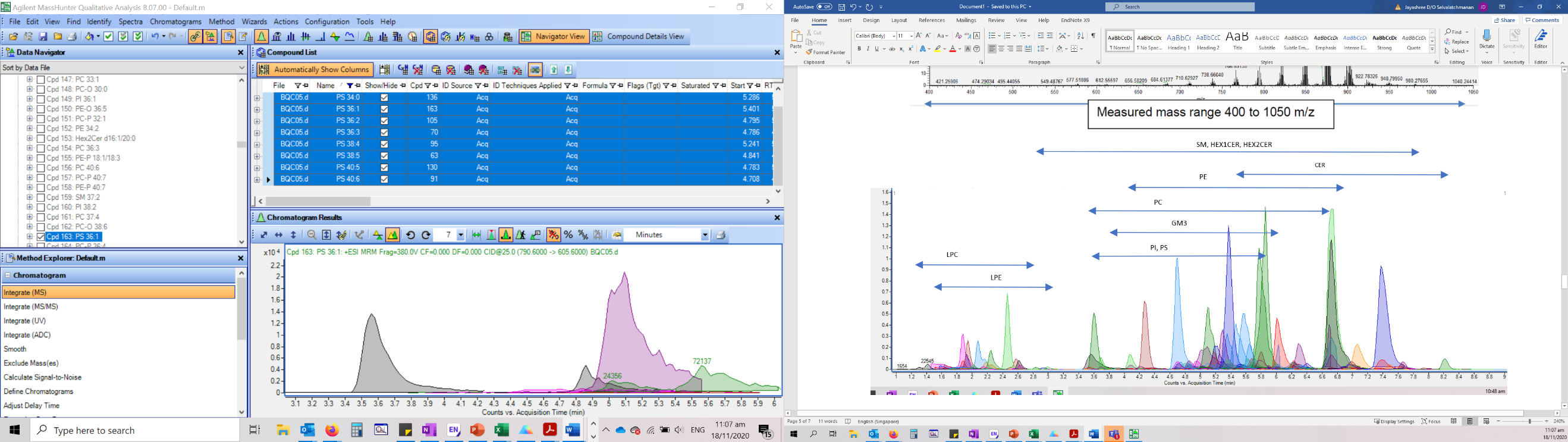


**Figure S4.** Representative chromatogram separating the various PL and SP classes via RP-LCMSMS (using dMRM)

**
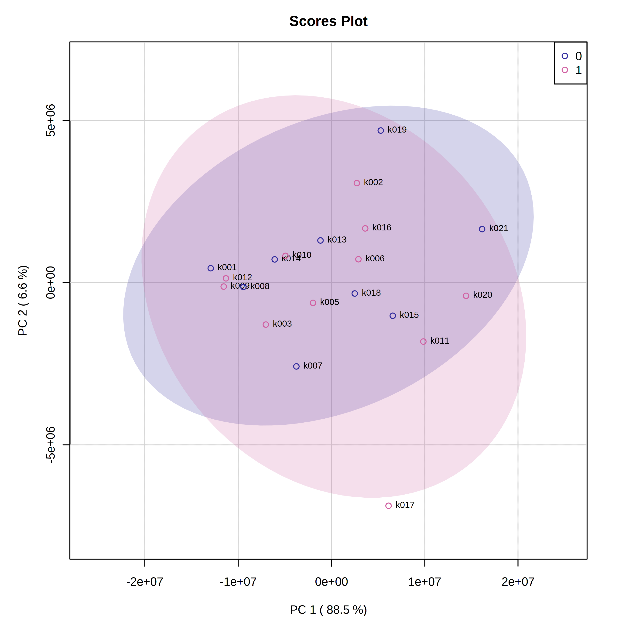
**

**Figure S5.** PCA plot of participants who provided a milk sample before (0) and after (1) dinner.

Table S1: Evening/Morning Concentration (Median) values for every PL and SL measured

| Lipid | Evening/Morning concentration (median) | Lipid | Evening/Morning concentration (median) |
| --- | --- | --- | --- |
| GM3 d18:1/16:0 | 1.17 | PC 32:1 | 1.21 |
| GM3 d18:1/18:0 | 1.19 | PC 32:2 | 1.10 |
| GM3 d18:1/20:0 | 1.41 | PC 33:0 | 1.18 |
| GM3 d18:1/22:0 | 1.15 | PC 33:1 | 1.36 |
| GM3 d18:1/24:0 | 1.08 | PC 34:0 | 1.11 |
| Cer d18:1/18:0 | 1.17 | PC 34:1 | 1.12 |
| Cer d18:1/20:0 | 1.21 | PC 34:2 | 1.18 |
| Cer d18:1/22:0 | 1.15 | PC 34:3 | 1.26 |
| Cer d18:1/23:0 | 1.24 | PC 35:2 | 1.26 |
| Cer d18:1/24:0 | 1.08 | PC 36:1 | 1.22 |
| Cer d18:1/24:1 | 1.10 | PC 36:2 | 1.23 |
| Cer d18:2/22:0 | 1.17 | PC 36:3 | 1.30 |
| Cer d18:2/24:0 | 1.09 | PC 36:4 | 1.19 |
| Hex1Cer d18:2/22:0 | 1.18 | PC 38:3 | 1.18 |
| Hex1Cer d18:2/24:0 | 1.09 | PC 38:4 | 1.12 |
| Hex2Cer d18:1/16:0 | 1.13 | PC 38:5 | 1.12 |
| Hex2Cer d18:1/20:0 | 1.20 | PC 38:6 | 1.09 |
| Hex2Cer d18:1/22:0 | 1.15 | PC 40:6 | 1.17 |
| Hex2Cer d18:1/24:0 | 1.16 | PC 40:8 | 1.32 |
| LPC 14:0 | 1.00 | PS 36:1 | 1.08 |
| LPC 18:1 | 1.14 | PS 36:2 | 1.12 |
| LPC 20:1 | 1.13 | PS 36:3 | 1.18 |
| LPC 20:3 | 1.07 | PS 38:4 | 1.11 |
| LPC 22:5 | 1.01 | PS 40:6 | 1.09 |
| LPC 22:6 | 0.97 | SM 30:1 | 0.99 |
| LPE 16:0 | 1.01 | SM 31:1 | 1.03 |
| LPE 18:0 | 1.24 | SM 32:0 | 1.02 |
| LPE 18:1 | 1.09 | SM 32:1 | 1.07 |
| LPE 20:1 | 1.10 | SM 33:1 | 1.07 |
| LPE 22:1 | 1.11 | SM 34:0 | 1.05 |
| LPE 22:5 | 1.10 | SM 34:1 | 1.06 |
| PE 32:0 | 1.35 | SM 34:2 | 1.02 |
| PE 34:0 | 1.25 | SM 35:1 | 1.16 |
| PE 34:1 | 1.32 | SM 36:0 | 1.14 |
| PE 36:1 | 1.39 | SM 36:1 | 1.07 |
| PE 36:2 | 1.30 | SM 36:2 | 1.14 |
| PE 36:4 | 1.48 | SM 37:1 | 1.07 |
| PE 38:1 | 1.23 | SM 38:0 | 1.08 |
| PE 38:2 | 1.36 | SM 38:1 | 1.09 |
| PE 38:3 | 1.35 | SM 38:2 | 1.01 |
| PE 38:4 | 1.33 | SM 39:1 | 1.05 |
| PE 38:6 | 1.50 | SM 40:0 | 1.07 |
| PE 40:2 | 1.32 | SM 40:1 | 1.08 |
| PE 40:7 | 1.34 | SM 40:2 | 1.02 |
| PI 34:1 | 1.18 | SM 40:3 | 1.02 |
| PI 34:2 | 1.10 | SM 41:1 | 1.09 |
| PI 36:1 | 1.21 | SM 42:0 | 1.05 |
| PI 36:2 | 1.08 | SM 42:1 | 1.04 |
| PI 36:3 | 1.13 | SM 42:2 | 1.05 |
| PI 38:3 | 1.13 | SM 42:3 | 0.99 |
| PI 38:4 | 1.21 | SM 43:1 | 1.13 |
| PI 38:5 | 1.22 | SM 43:2 | 1.02 |
| PC 30:0 | 1.12 | SM 44:1 | 1.03 |
| PC 31:0 | 1.24 | SM 44:2 | 1.11 |
| PC 32:0 | 1.08 |  |  |

Table S2: Evening/Morning Concentration (Median) values for every TAG and DAG measured

| Lipid | Evening/Morning concentration (median) | Lipid | Evening/Morning concentration (median) | Lipid | Evening/Morning concentration (median) |
| --- | --- | --- | --- | --- | --- |
| DAG 32:0 | 1.20 | TAG 47:4 | 1.15 | TAG 56:5 | 1.36 |
| DAG 34:2 | 1.09 | TAG 47:3 | 1.36 | TAG 56:4 | 1.32 |
| DAG 34:1 | 1.06 | TAG 47:2 | 1.39 | TAG 56:3 | 1.42 |
| DAG 34:0 | 1.17 | TAG 47:1 | 1.47 | TAG 56:2 | 1.50 |
| DAG 36:4 | 1.11 | TAG 48:7 | 1.37 | TAG 56:1 | 1.29 |
| DAG 36:3 | 1.12 | TAG 47:0 | 1.76 | TAG 58:10 | 1.17 |
| DAG 36:2 | 1.03 | TAG 48:6 | 1.26 | TAG 58:9 | 1.36 |
| DAG 36:1 | 0.95 | TAG 48:5 | 1.09 | TAG 58:8 | 1.31 |
| DAG 38:6 | 0.96 | TAG 48:4 | 1.10 | TAG 58:7 | 1.37 |
| DAG 44:10 | 1.25 | TAG 48:3 | 1.14 | TAG 58:6 | 1.36 |
| TAG 32:0 | 1.20 | TAG 48:2 | 1.12 | TAG 58:5 | 1.24 |
| TAG 34:1 | 1.64 | TAG 48:1 | 1.41 | TAG 58:4 | 1.24 |
| TAG 34:0 | 1.37 | TAG 49:4 | 1.53 |  |  |
| TAG 36:2 | 1.80 | TAG 49:3 | 1.43 |  |  |
| TAG 36:1 | 1.55 | TAG 49:2 | 1.53 |  |  |
| TAG 36:0 | 1.42 | TAG 50:8 | 1.22 |  |  |
| TAG 37:0 | 1.55 | TAG 49:1 | 1.88 |  |  |
| TAG 38:3 | 1.22 | TAG 50:7 | 1.15 |  |  |
| TAG 38:2 | 1.37 | TAG 50:6 | 1.25 |  |  |
| TAG 38:1 | 1.46 | TAG 50:5 | 1.25 |  |  |
| TAG 38:0 | 1.42 | TAG 50:4 | 1.22 |  |  |
| TAG 39:1 | 1.54 | TAG 50:3 | 1.28 |  |  |
| TAG 39:0 | 1.55 | TAG 50:2 | 1.27 |  |  |
| TAG 40:4 | 1.33 | TAG 50:1 | 1.34 |  |  |
| TAG 40:3 | 1.22 | TAG 51:4 | 1.61 |  |  |
| TAG 40:2 | 1.16 | TAG 51:3 | 1.60 |  |  |
| TAG 40:1 | 1.16 | TAG 51:2 | 1.66 |  |  |
| TAG 40:0 | 1.25 | TAG 52:8 | 1.09 |  |  |
| TAG 41:1 | 1.54 | TAG 51:1 | 1.68 |  |  |
| TAG 41:0 | 1.48 | TAG 52:7 | 1.12 |  |  |
| TAG 42:5 | 1.57 | TAG 52:6 | 1.30 |  |  |
| TAG 42:4 | 1.20 | TAG 52:5 | 1.42 |  |  |
| TAG 42:3 | 1.14 | TAG 52:4 | 1.41 |  |  |
| TAG 42:2 | 1.13 | TAG 52:3 | 1.36 |  |  |
| TAG 42:1 | 1.18 | TAG 52:2 | 1.39 |  |  |
| TAG 42:0 | 1.22 | TAG 53:5 | 1.48 |  |  |
| TAG 43:2 | 1.53 | TAG 53:4 | 1.45 |  |  |
| TAG 43:1 | 1.48 | TAG 53:3 | 1.52 |  |  |
| TAG 43:0 | 1.36 | TAG 53:2 | 1.65 |  |  |
| TAG 44:6 | 1.45 | TAG 54:8 | 1.37 |  |  |
| TAG 44:5 | 1.46 | TAG 53:1 | 1.60 |  |  |
| TAG 44:4 | 1.21 | TAG 54:7 | 1.54 |  |  |
| TAG 44:3 | 1.20 | TAG 54:6 | 1.48 |  |  |
| TAG 44:2 | 1.25 | TAG 54:5 | 1.34 |  |  |
| TAG 44:1 | 1.30 | TAG 54:4 | 1.39 |  |  |
| TAG 44:0 | 1.11 | TAG 54:3 | 1.35 |  |  |
| TAG 45:3 | 1.42 | TAG 54:2 | 1.37 |  |  |
| TAG 45:2 | 1.36 | TAG 55:6 | 1.78 |  |  |
| TAG 45:1 | 1.39 | TAG 55:5 | 1.62 |  |  |
| TAG 45:0 | 1.49 | TAG 55:4 | 1.50 |  |  |
| TAG 46:6 | 1.25 | TAG 55:3 | 1.62 |  |  |
| TAG 46:5 | 1.35 | TAG 56:9 | 1.28 |  |  |
| TAG 46:4 | 1.21 | TAG 55:2 | 1.78 |  |  |
| TAG 46:3 | 1.16 | TAG 56:8 | 1.35 |  |  |
| TAG 46:2 | 1.24 | TAG 56:7 | 1.31 |  |  |
| TAG 46:1 | 1.30 | TAG 56:6 | 1.34 |  |  |

Table S3: List of class-specific internal standards (ISTDs) made up in extraction solvent (MTBE/MeOH 7:2) and their final concentrations in the measured extract. DAG was normalised to TAG standard.


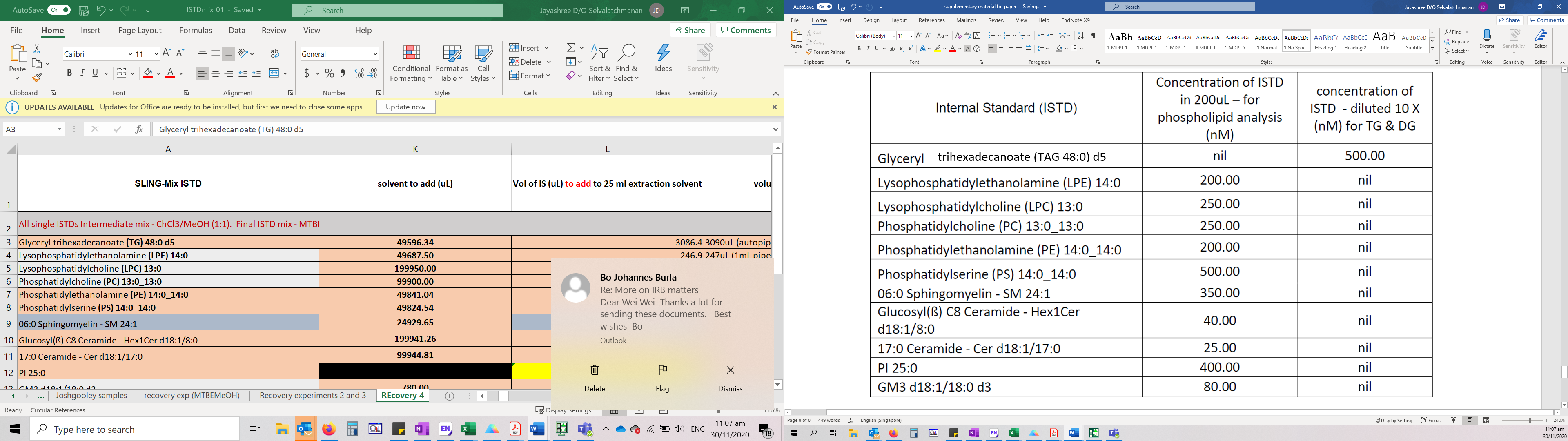


Table S4: Variance components analysis of individual lipids. Z: Wald Z-test value, p: p-value (significance set to 0.05), ICC: Intra-class correlation coefficient, F: F-test value.

| **Number** | **Analyte name** | **Variance analysis** | | | **Time of day** | | **Order of study visit** | |
| --- | --- | --- | --- | --- | --- | --- | --- | --- |
|  |  | **Z** | **p** | **ICC** | **F** | **p** | **F** | **p** |
| 1 | PE_34_1 | 2.73 | 0.00 | 0.80 | 20.29 | 0.00 | 2.39 | 0.14 |
| 2 | PE_32_0 | 2.80 | 0.00 | 0.84 | 17.37 | 0.00 | 8.26 | 0.01 |
| 3 | PE_38_3 | 2.44 | 0.01 | 0.68 | 16.26 | 0.00 | 2.74 | 0.12 |
| 4 | PE_38_2 | 2.79 | 0.00 | 0.83 | 15.75 | 0.00 | 3.46 | 0.08 |
| 5 | PE_36_1 | 2.38 | 0.01 | 0.65 | 14.20 | 0.00 | 3.85 | 0.07 |
| 6 | LPE_18_0 | 2.40 | 0.01 | 0.66 | 13.40 | 0.00 | 0.77 | 0.39 |
| 7 | PE_40_7 | 2.39 | 0.01 | 0.66 | 12.92 | 0.00 | 2.31 | 0.15 |
| 8 | PE_36_2 | 2.01 | 0.02 | 0.52 | 11.81 | 0.00 | 1.07 | 0.31 |
| 9 | PE_38_6 | 2.42 | 0.01 | 0.67 | 11.79 | 0.00 | 1.52 | 0.23 |
| 10 | PE_38_4 | 1.24 | 0.11 | 0.30 | 11.08 | 0.00 | 0.01 | 0.91 |
| 11 | PE_36_4 | 2.42 | 0.01 | 0.67 | 10.97 | 0.00 | 0.60 | 0.45 |
| 12 | TG_50_1 | 1.73 | 0.04 | 0.43 | 9.69 | 0.01 | 1.34 | 0.26 |
| 13 | TG_52_3 | 1.89 | 0.03 | 0.48 | 8.61 | 0.01 | 0.35 | 0.56 |
| 14 | TG_44_5 | 2.28 | 0.01 | 0.62 | 8.45 | 0.01 | 1.11 | 0.31 |
| 15 | TG_56_8 | 2.06 | 0.02 | 0.54 | 8.44 | 0.01 | 1.23 | 0.28 |
| 16 | TG_46_5 | 2.25 | 0.01 | 0.60 | 8.04 | 0.01 | 0.70 | 0.41 |
| 17 | TG_50_2 | 1.90 | 0.03 | 0.49 | 7.97 | 0.01 | 1.13 | 0.30 |
| 18 | TG_52_4 | 2.24 | 0.01 | 0.60 | 7.92 | 0.01 | 0.16 | 0.70 |
| 19 | TG_44_4 | 2.21 | 0.01 | 0.59 | 7.91 | 0.01 | 0.64 | 0.43 |
| 20 | TG_40_4 | 2.26 | 0.01 | 0.61 | 7.90 | 0.01 | 0.06 | 0.80 |
| 21 | TG_42_4 | 1.92 | 0.03 | 0.49 | 7.76 | 0.01 | 0.34 | 0.57 |
| 22 | SM_36_0 | 2.33 | 0.01 | 0.63 | 7.74 | 0.01 | 0.29 | 0.60 |
| 23 | TG_56_7 | 1.71 | 0.04 | 0.43 | 7.58 | 0.01 | 1.06 | 0.32 |
| 24 | TG_43_2 | 2.00 | 0.02 | 0.52 | 7.51 | 0.01 | 1.92 | 0.18 |
| 25 | PE_40_2 | 2.84 | 0.00 | 0.86 | 7.49 | 0.01 | 4.46 | 0.05 |
| 26 | TG_56_6 | 1.52 | 0.06 | 0.38 | 7.36 | 0.01 | 0.69 | 0.42 |
| 27 | TG_52_6 | 2.18 | 0.01 | 0.58 | 7.33 | 0.01 | 1.79 | 0.20 |
| 28 | TG_48_6 | 2.21 | 0.01 | 0.59 | 7.27 | 0.01 | 2.29 | 0.15 |
| 29 | TG_50_3 | 2.11 | 0.02 | 0.56 | 7.20 | 0.02 | 1.29 | 0.27 |
| 30 | TG_42_5 | 2.11 | 0.02 | 0.55 | 7.18 | 0.02 | 0.24 | 0.63 |
| 31 | TG_49_4 | 2.19 | 0.01 | 0.58 | 7.05 | 0.02 | 1.75 | 0.20 |
| 32 | PE_34_0 | 2.68 | 0.00 | 0.78 | 7.00 | 0.02 | 9.48 | 0.01 |
| 33 | TG_44_3 | 2.19 | 0.01 | 0.58 | 6.95 | 0.02 | 1.26 | 0.28 |
| 34 | TG_52_5 | 2.30 | 0.01 | 0.62 | 6.94 | 0.02 | 0.28 | 0.60 |
| 35 | TG_50_5 | 2.33 | 0.01 | 0.63 | 6.93 | 0.02 | 1.30 | 0.27 |
| 36 | TG_46_4 | 2.17 | 0.01 | 0.58 | 6.91 | 0.02 | 0.59 | 0.45 |
| 37 | TG_51_3 | 1.85 | 0.03 | 0.47 | 6.91 | 0.02 | 0.72 | 0.41 |
| 38 | TG_48_1 | 2.17 | 0.02 | 0.58 | 6.84 | 0.02 | 3.11 | 0.09 |
| 39 | TG_50_4 | 2.37 | 0.01 | 0.65 | 6.78 | 0.02 | 1.17 | 0.29 |
| 40 | TG_49_3 | 2.07 | 0.02 | 0.54 | 6.76 | 0.02 | 1.33 | 0.26 |
| 41 | TG_51_4 | 1.83 | 0.03 | 0.47 | 6.76 | 0.02 | 0.27 | 0.61 |
| 42 | TG_47_3 | 2.28 | 0.01 | 0.62 | 6.74 | 0.02 | 2.48 | 0.13 |
| 43 | TG_49_2 | 2.06 | 0.02 | 0.54 | 6.73 | 0.02 | 1.67 | 0.21 |
| 44 | TG_38_2 | 2.15 | 0.02 | 0.57 | 6.73 | 0.02 | 0.22 | 0.64 |
| 45 | TG_54_8 | 2.28 | 0.01 | 0.62 | 6.68 | 0.02 | 0.69 | 0.42 |
| 46 | TG_54_6 | 2.36 | 0.01 | 0.64 | 6.66 | 0.02 | 0.06 | 0.81 |
| 47 | TG_44_2 | 2.10 | 0.02 | 0.55 | 6.60 | 0.02 | 1.56 | 0.23 |
| 48 | TG_54_5 | 2.25 | 0.01 | 0.60 | 6.57 | 0.02 | 0.15 | 0.70 |
| 49 | TG_58_9 | 2.06 | 0.02 | 0.54 | 6.56 | 0.02 | 1.05 | 0.32 |
| 50 | TG_50_6 | 2.26 | 0.01 | 0.61 | 6.54 | 0.02 | 2.68 | 0.12 |
| 51 | PC_36_1 | 2.42 | 0.01 | 0.67 | 6.51 | 0.02 | 1.67 | 0.21 |
| 52 | PI_38_3 | 2.13 | 0.02 | 0.56 | 6.46 | 0.02 | 2.28 | 0.15 |
| 53 | TG_54_7 | 2.29 | 0.01 | 0.62 | 6.43 | 0.02 | 0.18 | 0.68 |
| 54 | TG_45_2 | 2.11 | 0.02 | 0.55 | 6.35 | 0.02 | 2.15 | 0.16 |
| 55 | TG_38_3 | 2.21 | 0.01 | 0.59 | 6.35 | 0.02 | 0.41 | 0.53 |
| 56 | GM3_d18_1_18_0 | 2.35 | 0.01 | 0.64 | 6.34 | 0.02 | 2.15 | 0.16 |
| 57 | TG_46_6 | 2.02 | 0.02 | 0.53 | 6.33 | 0.02 | 1.37 | 0.26 |
| 58 | TG_40_3 | 2.07 | 0.02 | 0.54 | 6.28 | 0.02 | 0.44 | 0.52 |
| 59 | TG_36_2 | 2.24 | 0.01 | 0.60 | 6.28 | 0.02 | 0.62 | 0.44 |
| 60 | TG_43_1 | 2.09 | 0.02 | 0.55 | 6.27 | 0.02 | 2.62 | 0.12 |
| 61 | TG_45_3 | 2.05 | 0.02 | 0.54 | 6.25 | 0.02 | 1.81 | 0.19 |
| 62 | TG_48_7 | 1.93 | 0.03 | 0.50 | 6.24 | 0.02 | 1.27 | 0.27 |
| 63 | GM3_d18_1_22_0 | 1.62 | 0.05 | 0.40 | 6.22 | 0.02 | 2.48 | 0.13 |
| 64 | PE_38_1 | 2.82 | 0.00 | 0.85 | 6.21 | 0.02 | 4.65 | 0.04 |
| 65 | PC_40_6 | 2.21 | 0.01 | 0.59 | 6.13 | 0.02 | 3.06 | 0.10 |
| 66 | TG_44_1 | 2.09 | 0.02 | 0.55 | 6.10 | 0.02 | 2.25 | 0.15 |
| 67 | TG_50_8 | 2.46 | 0.01 | 0.69 | 6.07 | 0.02 | 2.55 | 0.13 |
| 68 | TG_42_3 | 2.15 | 0.02 | 0.57 | 6.02 | 0.02 | 1.22 | 0.28 |
| 69 | DAG_44_10 | 2.13 | 0.02 | 0.56 | 6.01 | 0.02 | 0.80 | 0.38 |
| 70 | TG_44_6 | 1.92 | 0.03 | 0.49 | 5.93 | 0.03 | 0.85 | 0.37 |
| 71 | TG_56_5 | 1.62 | 0.05 | 0.40 | 5.83 | 0.03 | 0.93 | 0.35 |
| 72 | TG_46_3 | 2.18 | 0.01 | 0.58 | 5.80 | 0.03 | 1.57 | 0.23 |
| 73 | TG_56_9 | 2.23 | 0.01 | 0.60 | 5.80 | 0.03 | 1.38 | 0.25 |
| 74 | PS_38_4 | 0.69 | 0.24 | 0.16 | 5.76 | 0.03 | 0.39 | 0.54 |
| 75 | TG_49_1 | 2.00 | 0.02 | 0.52 | 5.75 | 0.03 | 2.15 | 0.16 |
| 76 | PC_36_3 | 2.05 | 0.02 | 0.53 | 5.68 | 0.03 | 2.00 | 0.17 |
| 77 | TG_48_5 | 2.54 | 0.01 | 0.72 | 5.64 | 0.03 | 2.67 | 0.12 |
| 78 | TG_37_0 | 1.57 | 0.06 | 0.39 | 5.54 | 0.03 | 2.32 | 0.15 |
| 79 | TG_54_4 | 1.84 | 0.03 | 0.47 | 5.53 | 0.03 | 0.44 | 0.52 |
| 80 | TG_39_0 | 2.16 | 0.02 | 0.57 | 5.53 | 0.03 | 4.44 | 0.05 |
| 81 | TG_46_1 | 2.21 | 0.01 | 0.59 | 5.50 | 0.03 | 2.84 | 0.11 |
| 82 | TG_53_5 | 1.86 | 0.03 | 0.47 | 5.50 | 0.03 | 0.35 | 0.56 |
| 83 | TG_41_1 | 1.86 | 0.03 | 0.47 | 5.44 | 0.03 | 1.94 | 0.18 |
| 84 | TG_50_7 | 2.22 | 0.01 | 0.59 | 5.42 | 0.03 | 1.72 | 0.21 |
| 85 | TG_47_2 | 2.21 | 0.01 | 0.59 | 5.42 | 0.03 | 2.84 | 0.11 |
| 86 | PI_36_1 | 2.23 | 0.01 | 0.60 | 5.39 | 0.03 | 3.45 | 0.08 |
| 87 | TG_40_2 | 2.02 | 0.02 | 0.52 | 5.39 | 0.03 | 0.65 | 0.43 |
| 88 | TG_51_2 | 1.76 | 0.04 | 0.44 | 5.38 | 0.03 | 1.09 | 0.31 |
| 89 | SM_40_0 | 2.15 | 0.02 | 0.57 | 5.28 | 0.03 | 0.73 | 0.40 |
| 90 | TG_55_5 | 1.39 | 0.08 | 0.34 | 5.28 | 0.03 | 0.86 | 0.37 |
| 91 | PI_36_3 | 2.46 | 0.01 | 0.68 | 5.22 | 0.03 | 0.35 | 0.56 |
| 92 | TG_58_8 | 1.70 | 0.04 | 0.43 | 5.21 | 0.03 | 0.81 | 0.38 |
| 93 | TG_53_4 | 1.74 | 0.04 | 0.44 | 5.18 | 0.04 | 0.45 | 0.51 |
| 94 | TG_52_7 | 2.28 | 0.01 | 0.61 | 5.18 | 0.04 | 2.70 | 0.12 |
| 95 | PS_36_3 | 2.46 | 0.01 | 0.68 | 5.17 | 0.04 | 1.57 | 0.23 |
| 96 | PI_36_2 | 2.28 | 0.01 | 0.62 | 5.13 | 0.04 | 0.49 | 0.49 |
| 97 | PC_32_0 | 2.32 | 0.01 | 0.63 | 5.12 | 0.04 | 2.29 | 0.15 |
| 98 | TG_45_1 | 2.19 | 0.01 | 0.58 | 5.11 | 0.04 | 2.99 | 0.10 |
| 99 | TG_48_2 | 2.18 | 0.01 | 0.58 | 5.09 | 0.04 | 2.66 | 0.12 |
| 100 | TG_38_1 | 1.75 | 0.04 | 0.44 | 5.08 | 0.04 | 0.25 | 0.62 |
| 101 | PI_34_2 | 2.28 | 0.01 | 0.62 | 5.06 | 0.04 | 0.87 | 0.36 |
| 102 | Hex2Cer_d18_1_24_0 | 2.35 | 0.01 | 0.64 | 4.98 | 0.04 | 0.42 | 0.52 |
| 103 | TG_46_2 | 2.21 | 0.01 | 0.59 | 4.94 | 0.04 | 2.32 | 0.15 |
| 104 | TG_42_0 | 2.11 | 0.02 | 0.56 | 4.93 | 0.04 | 4.05 | 0.06 |
| 105 | TG_55_6 | 1.55 | 0.06 | 0.38 | 4.93 | 0.04 | 1.40 | 0.25 |
| 106 | PC_35_2 | 2.32 | 0.01 | 0.63 | 4.84 | 0.04 | 1.09 | 0.31 |
| 107 | TG_58_10 | 2.32 | 0.01 | 0.63 | 4.84 | 0.04 | 0.65 | 0.43 |
| 108 | TG_47_0 | 2.33 | 0.01 | 0.64 | 4.83 | 0.04 | 2.75 | 0.11 |
| 109 | TG_47_1 | 2.24 | 0.01 | 0.60 | 4.78 | 0.04 | 3.45 | 0.08 |
| 110 | TG_36_0 | 1.90 | 0.03 | 0.48 | 4.74 | 0.04 | 1.54 | 0.23 |
| 111 | TG_41_0 | 2.26 | 0.01 | 0.61 | 4.73 | 0.04 | 4.22 | 0.05 |
| 112 | TG_58_7 | 1.30 | 0.10 | 0.32 | 4.66 | 0.04 | 0.73 | 0.41 |
| 113 | TG_40_1 | 1.90 | 0.03 | 0.49 | 4.62 | 0.05 | 0.91 | 0.35 |
| 114 | TG_51_1 | 1.67 | 0.05 | 0.42 | 4.52 | 0.05 | 1.25 | 0.28 |
| 115 | TG_34_0 | 2.00 | 0.02 | 0.52 | 4.46 | 0.05 | 1.60 | 0.22 |
| 116 | Hex1Cer_d18_2_22_0 | 2.22 | 0.01 | 0.59 | 4.44 | 0.05 | 0.07 | 0.79 |
| 117 | PC_36_4 | 2.54 | 0.01 | 0.72 | 4.44 | 0.05 | 1.38 | 0.26 |
| 118 | TG_38_0 | 1.94 | 0.03 | 0.50 | 4.42 | 0.05 | 2.32 | 0.15 |
| 119 | TG_32_0 | 2.02 | 0.02 | 0.53 | 4.42 | 0.05 | 1.27 | 0.27 |
| 120 | PI_34_1 | 2.32 | 0.01 | 0.63 | 4.38 | 0.05 | 0.52 | 0.48 |
| 121 | PS_36_1 | 2.47 | 0.01 | 0.69 | 4.38 | 0.05 | 0.29 | 0.60 |
| 122 | TG_45_0 | 2.35 | 0.01 | 0.64 | 4.36 | 0.05 | 3.69 | 0.07 |
| 123 | PC_40_8 | 2.62 | 0.00 | 0.75 | 4.36 | 0.05 | 1.57 | 0.23 |
| 124 | TG_42_2 | 2.15 | 0.02 | 0.57 | 4.30 | 0.05 | 1.55 | 0.23 |
| 125 | TG_36_1 | 1.75 | 0.04 | 0.44 | 4.28 | 0.05 | 0.70 | 0.41 |
| 126 | PC_36_2 | 2.21 | 0.01 | 0.59 | 4.27 | 0.05 | 1.72 | 0.21 |
| 127 | TG_43_0 | 2.24 | 0.01 | 0.60 | 4.26 | 0.05 | 3.56 | 0.08 |
| 128 | GM3_d18_1_16_0 | 2.46 | 0.01 | 0.69 | 4.15 | 0.06 | 3.94 | 0.06 |
| 129 | TG_55_4 | 1.57 | 0.06 | 0.39 | 4.14 | 0.06 | 1.01 | 0.33 |
| 130 | PC_38_3 | 2.26 | 0.01 | 0.61 | 4.13 | 0.06 | 0.76 | 0.39 |
| 131 | TG_58_6 | 1.11 | 0.13 | 0.27 | 4.07 | 0.06 | 0.60 | 0.45 |
| 132 | GM3_d18_1_20_0 | 0.93 | 0.18 | 0.22 | 4.01 | 0.06 | 1.46 | 0.24 |
| 133 | PC_33_1 | 2.51 | 0.01 | 0.71 | 3.98 | 0.06 | 1.05 | 0.32 |
| 134 | PI_38_4 | 1.75 | 0.04 | 0.44 | 3.97 | 0.06 | 1.23 | 0.28 |
| 135 | SM_42_0 | 2.45 | 0.01 | 0.68 | 3.95 | 0.06 | 0.39 | 0.54 |
| 136 | TG_44_0 | 2.11 | 0.02 | 0.55 | 3.91 | 0.06 | 3.61 | 0.07 |
| 137 | TG_52_8 | 2.33 | 0.01 | 0.63 | 3.91 | 0.06 | 2.68 | 0.12 |
| 138 | TG_42_1 | 1.97 | 0.02 | 0.51 | 3.89 | 0.06 | 1.58 | 0.22 |
| 139 | TG_48_3 | 2.27 | 0.01 | 0.61 | 3.87 | 0.06 | 2.00 | 0.17 |
| 140 | TG_53_3 | 1.48 | 0.07 | 0.36 | 3.85 | 0.07 | 0.68 | 0.42 |
| 141 | TG_39_1 | 1.39 | 0.08 | 0.34 | 3.83 | 0.07 | 0.84 | 0.37 |
| 142 | PC_38_4 | 1.30 | 0.10 | 0.31 | 3.81 | 0.07 | 0.87 | 0.36 |
| 143 | Cer_d18_2_22_0 | 2.18 | 0.01 | 0.58 | 3.75 | 0.07 | 0.15 | 0.71 |
| 144 | Hex2Cer_d18_1_22_0 | 2.20 | 0.01 | 0.59 | 3.70 | 0.07 | 0.09 | 0.77 |
| 145 | TG_40_0 | 2.02 | 0.02 | 0.52 | 3.64 | 0.07 | 3.39 | 0.08 |
| 146 | PS_36_2 | 1.83 | 0.03 | 0.47 | 3.64 | 0.07 | 0.93 | 0.35 |
| 147 | PC_34_3 | 2.53 | 0.01 | 0.71 | 3.54 | 0.08 | 1.35 | 0.26 |
| 148 | SM_44_2 | 2.87 | 0.00 | 0.88 | 3.54 | 0.08 | 1.72 | 0.21 |
| 149 | SM_36_2 | 1.83 | 0.03 | 0.47 | 3.49 | 0.08 | 0.03 | 0.85 |
| 150 | TG_55_2 | 1.59 | 0.06 | 0.39 | 3.47 | 0.08 | 2.69 | 0.12 |
| 151 | TG_34_1 | 1.79 | 0.04 | 0.45 | 3.44 | 0.08 | 1.00 | 0.33 |
| 152 | SM_38_1 | 1.65 | 0.05 | 0.41 | 3.43 | 0.08 | 1.20 | 0.29 |
| 153 | TG_53_1 | 1.67 | 0.05 | 0.42 | 3.32 | 0.08 | 1.96 | 0.18 |
| 154 | TG_48_4 | 2.51 | 0.01 | 0.70 | 3.26 | 0.09 | 2.08 | 0.17 |
| 155 | PC_34_2 | 1.81 | 0.03 | 0.46 | 3.26 | 0.09 | 0.29 | 0.60 |
| 156 | PI_38_5 | 2.52 | 0.01 | 0.71 | 3.23 | 0.09 | 2.59 | 0.12 |
| 157 | TG_47_4 | 1.99 | 0.02 | 0.52 | 3.19 | 0.09 | 0.05 | 0.83 |
| 158 | SM_38_0 | 1.79 | 0.04 | 0.45 | 3.11 | 0.09 | 0.52 | 0.48 |
| 159 | PC_33_0 | 2.66 | 0.00 | 0.77 | 3.03 | 0.10 | 2.99 | 0.10 |
| 160 | LPE_22_5 | 2.57 | 0.01 | 0.73 | 3.03 | 0.10 | 0.29 | 0.60 |
| 161 | TG_58_5 | 1.23 | 0.11 | 0.30 | 2.99 | 0.10 | 0.94 | 0.35 |
| 162 | SM_34_0 | 2.34 | 0.01 | 0.64 | 2.97 | 0.10 | 0.20 | 0.66 |
| 163 | TG_55_3 | 1.43 | 0.08 | 0.35 | 2.96 | 0.10 | 1.37 | 0.26 |
| 164 | PC_34_1 | 2.33 | 0.01 | 0.64 | 2.84 | 0.11 | 1.68 | 0.21 |
| 165 | TG_54_3 | 1.39 | 0.08 | 0.34 | 2.82 | 0.11 | 0.85 | 0.37 |
| 166 | SM_36_1 | 1.91 | 0.03 | 0.49 | 2.79 | 0.11 | 0.06 | 0.82 |
| 167 | Hex1Cer_d18_2_24_0 | 2.57 | 0.01 | 0.73 | 2.75 | 0.11 | 1.73 | 0.21 |
| 168 | GM3_d18_1_24_0 | 1.97 | 0.02 | 0.51 | 2.70 | 0.12 | 0.46 | 0.50 |
| 169 | TG_58_1 | 2.19 | 0.01 | 0.58 | 2.69 | 0.12 | 3.01 | 0.10 |
| 170 | TG_56_1 | 1.55 | 0.06 | 0.38 | 2.67 | 0.12 | 2.24 | 0.15 |
| 171 | SM_39_1 | 1.99 | 0.02 | 0.51 | 2.67 | 0.12 | 0.01 | 0.91 |
| 172 | Hex2Cer_d18_1_20_0 | 2.51 | 0.01 | 0.70 | 2.64 | 0.12 | 1.94 | 0.18 |
| 173 | Cer_d18_1_22_0 | 2.16 | 0.02 | 0.57 | 2.61 | 0.12 | 1.17 | 0.29 |
| 174 | LPE_22_1 | 2.99 | 0.00 | 0.94 | 2.47 | 0.13 | 3.05 | 0.10 |
| 175 | LPE_20_1 | 2.77 | 0.00 | 0.82 | 2.45 | 0.13 | 1.37 | 0.26 |
| 176 | PC_38_6 | 2.41 | 0.01 | 0.67 | 2.42 | 0.14 | 0.00 | 1.00 |
| 177 | TG_53_2 | 1.43 | 0.08 | 0.35 | 2.40 | 0.14 | 0.94 | 0.35 |
| 178 | Cer_d18_1_24_0 | 2.48 | 0.01 | 0.69 | 2.33 | 0.14 | 1.30 | 0.27 |
| 179 | PS_40_6 | 2.45 | 0.01 | 0.68 | 2.33 | 0.14 | 0.58 | 0.46 |
| 180 | LPE_18_1 | 2.50 | 0.01 | 0.70 | 2.31 | 0.15 | 0.25 | 0.62 |
| 181 | TG_56_4 | 1.59 | 0.06 | 0.39 | 2.28 | 0.15 | 1.89 | 0.19 |
| 182 | SM_40_1 | 1.76 | 0.04 | 0.44 | 2.26 | 0.15 | 0.68 | 0.42 |
| 183 | SM_42_2 | 2.71 | 0.00 | 0.79 | 2.26 | 0.15 | 1.21 | 0.29 |
| 184 | SM_43_1 | 2.44 | 0.01 | 0.68 | 2.22 | 0.15 | 0.78 | 0.39 |
| 185 | LPC_20_1 | 2.79 | 0.00 | 0.83 | 2.16 | 0.16 | 1.55 | 0.23 |
| 186 | TG_58_2 | 1.56 | 0.06 | 0.39 | 2.16 | 0.16 | 3.39 | 0.08 |
| 187 | SM_37_1 | 1.48 | 0.07 | 0.36 | 2.14 | 0.16 | 0.12 | 0.74 |
| 188 | DAG_32_0 | 2.34 | 0.01 | 0.64 | 2.12 | 0.16 | 1.44 | 0.25 |
| 189 | SM_42_1 | 2.17 | 0.02 | 0.57 | 1.88 | 0.19 | 1.12 | 0.30 |
| 190 | LPC_18_1 | 2.53 | 0.01 | 0.71 | 1.84 | 0.19 | 0.23 | 0.64 |
| 191 | PC_32_1 | 2.38 | 0.01 | 0.65 | 1.74 | 0.20 | 2.20 | 0.15 |
| 192 | Cer_d18_1_20_0 | 1.85 | 0.03 | 0.47 | 1.70 | 0.21 | 0.55 | 0.47 |
| 193 | SM_38_2 | 1.58 | 0.06 | 0.39 | 1.67 | 0.21 | 0.36 | 0.56 |
| 194 | PC_31_0 | 2.62 | 0.00 | 0.75 | 1.66 | 0.21 | 0.36 | 0.55 |
| 195 | TG_58_3 | 1.54 | 0.06 | 0.38 | 1.63 | 0.22 | 4.46 | 0.05 |
| 196 | PC_34_0 | 2.11 | 0.02 | 0.55 | 1.61 | 0.22 | 2.70 | 0.12 |
| 197 | SM_35_1 | 2.15 | 0.02 | 0.57 | 1.60 | 0.22 | 0.56 | 0.46 |
| 198 | Cer_d18_1_23_0 | 2.19 | 0.01 | 0.58 | 1.55 | 0.23 | 0.89 | 0.36 |
| 199 | DAG_34_1 | 2.53 | 0.01 | 0.71 | 1.50 | 0.24 | 1.55 | 0.23 |
| 200 | SM_41_1 | 1.98 | 0.02 | 0.51 | 1.49 | 0.24 | 0.26 | 0.62 |
| 201 | SM_43_2 | 2.83 | 0.00 | 0.85 | 1.37 | 0.26 | 0.47 | 0.50 |
| 202 | DAG_34_0 | 2.53 | 0.01 | 0.71 | 1.36 | 0.26 | 1.49 | 0.24 |
| 203 | TG_60_3 | 1.50 | 0.07 | 0.37 | 1.30 | 0.27 | 4.09 | 0.06 |
| 204 | TG_58_4 | 1.90 | 0.03 | 0.49 | 1.29 | 0.27 | 2.96 | 0.10 |
| 205 | TG_56_3 | 1.23 | 0.11 | 0.30 | 1.27 | 0.27 | 2.90 | 0.11 |
| 206 | TG_56_2 | 1.36 | 0.09 | 0.33 | 1.26 | 0.28 | 4.35 | 0.05 |
| 207 | SM_31_1 | 2.61 | 0.00 | 0.75 | 1.21 | 0.29 | 0.63 | 0.44 |
| 208 | Cer_d18_2_24_0 | 2.46 | 0.01 | 0.69 | 1.18 | 0.29 | 1.73 | 0.21 |
| 209 | Hex2Cer_d18_1_16_0 | 0.27 | 0.39 | 0.06 | 1.18 | 0.29 | 0.85 | 0.37 |
| 210 | SM_40_2 | 2.32 | 0.01 | 0.63 | 1.14 | 0.30 | 0.09 | 0.77 |
| 211 | DAG_34_2 | 2.62 | 0.00 | 0.75 | 1.12 | 0.30 | 1.35 | 0.26 |
| 212 | SM_32_1 | 2.51 | 0.01 | 0.70 | 1.10 | 0.31 | 0.86 | 0.36 |
| 213 | SM_44_1 | 2.47 | 0.01 | 0.69 | 1.07 | 0.31 | 0.57 | 0.46 |
| 214 | PC_38_5 | 2.21 | 0.01 | 0.59 | 1.04 | 0.32 | 0.98 | 0.33 |
| 215 | DAG_38_6 | 2.61 | 0.00 | 0.75 | 1.03 | 0.32 | 2.53 | 0.13 |
| 216 | PC_32_2 | 2.17 | 0.02 | 0.58 | 0.92 | 0.35 | 3.45 | 0.08 |
| 217 | SM_34_1 | 2.24 | 0.01 | 0.60 | 0.89 | 0.36 | 0.00 | 0.95 |
| 218 | SM_32_0 | 2.46 | 0.01 | 0.68 | 0.87 | 0.36 | 0.85 | 0.37 |
| 219 | DAG_36_1 | 2.54 | 0.01 | 0.72 | 0.82 | 0.38 | 1.94 | 0.18 |
| 220 | Cer_d18_1_18_0 | 2.19 | 0.01 | 0.58 | 0.78 | 0.39 | 1.17 | 0.29 |
| 221 | SM_42_3 | 2.37 | 0.01 | 0.65 | 0.77 | 0.39 | 0.18 | 0.68 |
| 222 | SM_33_1 | 2.51 | 0.01 | 0.71 | 0.65 | 0.43 | 1.12 | 0.30 |
| 223 | DAG_36_2 | 2.74 | 0.00 | 0.81 | 0.52 | 0.48 | 1.25 | 0.28 |
| 224 | TG_54_2 | 1.03 | 0.15 | 0.25 | 0.49 | 0.49 | 1.93 | 0.18 |
| 225 | PC_30_0 | 2.23 | 0.01 | 0.60 | 0.47 | 0.50 | 2.59 | 0.12 |
| 226 | LPC_20_3 | 2.48 | 0.01 | 0.69 | 0.45 | 0.51 | 0.38 | 0.55 |
| 227 | SM_34_2 | 2.45 | 0.01 | 0.68 | 0.40 | 0.53 | 0.02 | 0.88 |
| 228 | SM_40_3 | 2.06 | 0.02 | 0.54 | 0.40 | 0.53 | 0.20 | 0.66 |
| 229 | Cer_d18_1_24_1 | 2.46 | 0.01 | 0.68 | 0.38 | 0.54 | 1.90 | 0.18 |
| 230 | SM_30_1 | 2.31 | 0.01 | 0.63 | 0.28 | 0.60 | 0.51 | 0.48 |
| 231 | DAG_36_3 | 2.78 | 0.00 | 0.83 | 0.19 | 0.67 | 0.81 | 0.38 |
| 232 | LPE_16_0 | 2.82 | 0.00 | 0.85 | 0.17 | 0.69 | 1.27 | 0.27 |
| 233 | LPC_22_6 | 2.66 | 0.00 | 0.77 | 0.14 | 0.71 | 0.41 | 0.53 |
| 234 | LPC_14_0 | 2.46 | 0.01 | 0.69 | 0.03 | 0.87 | 1.27 | 0.28 |
| 235 | DAG_36_4 | 2.68 | 0.00 | 0.78 | 0.01 | 0.92 | 0.24 | 0.63 |
| 236 | LPC_22_5 | 2.42 | 0.01 | 0.67 | 0.01 | 0.93 | 0.15 | 0.71 |
| 237 | TG_52_2 | 1.82 | 0.03 | 0.49 | NaN | NaN | 0.49 | 0.49 |
